# Clinical recommendations for cardiovascular magnetic resonance mapping of T1, T2, T2* and extracellular volume: A consensus statement by the Society for Cardiovascular Magnetic Resonance (SCMR) endorsed by the European Association for Cardiovascular Imaging (EACVI)

**DOI:** 10.1186/s12968-017-0389-8

**Published:** 2017-10-09

**Authors:** Daniel R. Messroghli, James C. Moon, Vanessa M. Ferreira, Lars Grosse-Wortmann, Taigang He, Peter Kellman, Julia Mascherbauer, Reza Nezafat, Michael Salerno, Erik B. Schelbert, Andrew J. Taylor, Richard Thompson, Martin Ugander, Ruud B. van Heeswijk, Matthias G. Friedrich

**Affiliations:** 1 Department of Internal Medicine and Cardiology, Deutsches Herzzentrum Berlin, Berlin, Germany; 2 Department of Internal Medicine and Cardiology, Charité Universitätsmedizin Berlin, Augustenburger Platz 1, 13353 Berlin, Germany; 3 DZHK (German Centre for Cardiovascular Research), partner site Berlin, Augustenburger Platz 1, 13353 Berlin, Germany; 4 University College London and Barts Heart Centre, London, UK; 5 Oxford Centre for Clinical Magnetic Resonance Research, Division of Cardiovascular Medicine, Radcliffe Department of Medicine, University of Oxford, Oxford, UK; 6 Division of Cardiology in the Department of Pediatrics, The Hospital for Sick Children, University of Toronto, Toronto, ON Canada; 7 Cardiovascular Science Research Centre, St George’s, University of London, London, UK; 8 National Institutes of Health, Bethesda, MD USA; 9 Department of Internal Medicine II, Division of Cardiology, Vienna, Austria; 10 Department of Medicine (Cardiovascular Division), Beth Israel Deaconess Medical Center, Harvard Medical School, Boston, USA; 11 Departments of Medicine Cardiology Division, Radiology and Medical Imaging, and Biomedical Engineering, University of Virginia Health System, Charlottesville, VA USA; 12 Department of Medicine, University of Pittsburgh School of Medicine, Pittsburgh, PA USA; 13 UPMC Cardiovascular Magnetic Resonance Center, Heart and Vascular Institute, Pittsburgh, PA USA; 14 Clinical and Translational Science Institute, University of Pittsburgh, Pittsburgh, PA USA; 15 The Alfred Hospital, Baker Heart and Diabetes Institute, Melbourne, Australia; 16 Department of Biomedical Engineering, University of Alberta, Edmonton, Canada; 17 Department of Clinical Physiology, Karolinska Institutet, Karolinska University Hospital, Stockholm, Sweden; 18 Department of Radiology, Lausanne University Hospital (CHUV) and Lausanne University (UNIL), Lausanne, Switzerland; 19 Departments of Medicine and Diagnostic Radiology, McGill University, Montréal, Québec Canada; 20 Department of Medicine, Heidelberg University, Heidelberg, Germany; 21 Département de radiologie, Université de Montréal, Montréal, Québec Canada; 22 Departments of Cardiac Sciences and Radiology, University of Calgary, Calgary, Canada

## Abstract

Parametric mapping techniques provide a non-invasive tool for quantifying tissue alterations in myocardial disease in those eligible for cardiovascular magnetic resonance (CMR). Parametric mapping with CMR now permits the routine spatial visualization and quantification of changes in myocardial composition based on changes in T1, T2, and T2*(star) relaxation times and extracellular volume (ECV). These changes include specific disease pathways related to mainly *intracellular* disturbances of the cardiomyocyte (e.g., iron overload, or glycosphingolipid accumulation in Anderson-Fabry disease); *extracellular* disturbances in the myocardial interstitium (e.g., myocardial fibrosis or cardiac amyloidosis from accumulation of collagen or amyloid proteins, respectively); or both (myocardial edema with increased intracellular and/or extracellular water). Parametric mapping promises improvements in patient care through advances in quantitative diagnostics, inter- and intra-patient comparability, and relatedly improvements in treatment. There is a multitude of technical approaches and potential applications. This document provides a summary of the existing evidence for the clinical value of parametric mapping in the heart as of mid 2017, and gives recommendations for practical use in different clinical scenarios for scientists, clinicians, and CMR manufacturers.

## Background

CMR is the primary imaging modality for myocardial tissue characterization. CMR parametric mapping now permits the routine spatial visualization of quantitative changes in myocardium based on changes in myocardial parameters T1, T2, T2*(star) and ECV. These changes include specific disease pathways related to mainly *intracellular* disturbances of the cardiomyocyte (e.g., iron overload, or glycosphingolipid accumulation in Anderson-Fabry disease); *extracellular* disturbances in the myocardial interstitium (e.g., myocardial fibrosis of cardiac amyloidosis from accumulation of collagen or amyloid proteins, respectively); or both (e.g. myocardial edema and/or infarction with increased intracellular and/or extracellular water). Unlike T1-, T2-, or T2*-*weighted* images, mapping permits both visualization and quantification of the disease process, independent of whether myocardial disease is focal or diffuse. This innovation is important because historically, diffuse myocardial disease related to specific disease pathways has been difficult to non-invasively quantify or even appreciate.

Our technical capabilities with parametric mapping may exceed our understanding of how this data can guide optimal treatment for patients with signs or symptoms of underlying disease. Nonetheless, there is an important precedent demonstrating that image guided care exploiting CMR parametric measurements can improve patient outcome in iron overload states [1]. Thus, advances in CMR parametric mapping promise to improve patient care though better diagnostic decision-making, which in turn can result in better treatment, as a major step towards *Precision Medicine*. In addition, CMR parametric mapping also promises to facilitate the development of novel therapeutics, by providing quantitative endpoints reflective of the disease pathway of interest, especially in phase 2 efficacy trials. Technological advances now permit routine acquisition of parametric maps in patients eligible for CMR. While mapping adds unique and relevant diagnostic information on the status of the myocardium, its clinical application requires specific hardware, software, data acquisition and evaluation procedures, which are not completely standardized.

### Aims and scope of this document

This document provides recommendations for clinical and research applications of CMR myocardial T1, T2, T2*, and ECV mapping. We cite published evidence when available and provide expert consensus where incomplete. We recognize a priori that multiple methodologies for CMR parametric mapping do and should exist, with continued evolution and residual imperfections. Despite these limitations, abundant evidence demonstrates that parametric mapping appears robust under many conditions in its present form. We make analogy to another key cardiac imaging biomarker, the left ventricular ejection fraction (LVEF), where measurement variations persist within and across modalities, yet the yield of biological information is sufficient to diagnose disease, guide and monitor treatment, and to predict outcome. CMR parametric mapping goes beyond nonspecific functional surrogate markers of cardiovascular disease such as LVEF. Rather, CMR parametric mapping offers the potential to examine specific disease pathways that affect myocardial tissue composition.

In 2013, the “T1 Mapping Development Group” published a consensus statement that proposed suitable terminology and specific recommendations for site preparation, scan types, scan planning and acquisition, quality control, visualization and analysis, and technical directions [2]. Building on this initiative, the *Consensus Group on Cardiac MR Mapping* has formed itself to provide guidance on CMR mapping to scientists, clinicians, and manufacturers. The team includes experts with a wide and representative range of technical and clinical expertise, a broad geographical base and a balanced spectrum of interest.

Considering the rapidly increasing interest in mapping-based myocardial tissue characterization, the group developed this document to provide 1) an update on the available experimental and clinical evidence, 2) an updated list of clinical indications, 3) practical recommendations for state-of-the-art protocols and techniques, and 4) guidance for research.

## Terminology

Table 1 provides definitions for terminology related to the field of parametric mapping of the heart. We now recommend that ECV be expressed as a percentage (e.g. 25% rather than 0.25).

**Table 1:** Definitions of technical terms in the field of parametric mapping of the heart

Term	Meaning
T1 [ms]	Time constant representing the recovery of longitudinal magnetization (spin–lattice relaxation)
Native T1	T1 in the absence of an exogenous contrast agent
T2 [ms]	Time constant representing the decay of transverse magnetization (spin-spin relaxation)
T2* [ms]	Time constant representing the decay of transverse magnetization in the presence of local field inhomogeneities
ECV [%]	Extracellular volume fraction, calculated by $ECV=\frac{\left(\frac{1}{T1 my{o}_{postGd}}-\frac{1}{T1 my{o}_{native}}\right)}{\left(\frac{1}{T1 bloo{d}_{postGd}}-\frac{1}{T1 bloo{d}_{native}}\right)}\times \left(100- hematocrit\right)$ where myo = myocardium; blood = intracavitary blood pool; hematocrit = cellular volume fraction of blood [%]
Synthetic ECV [%]	ECV where hematocrit is not measured by laboratory blood sampling but derived from blood T1
Parametric mapping	A process where a secondary image is generated in which each pixel represents a specific magnetic tissue property (T1, T2, or T2*) or a derivative such as ECV) derived from the spatially corresponding voxel of a set of co-registered magnetic resonance source images

## Recommendations part I: Clinical indications and utility

**Fig. 1:**
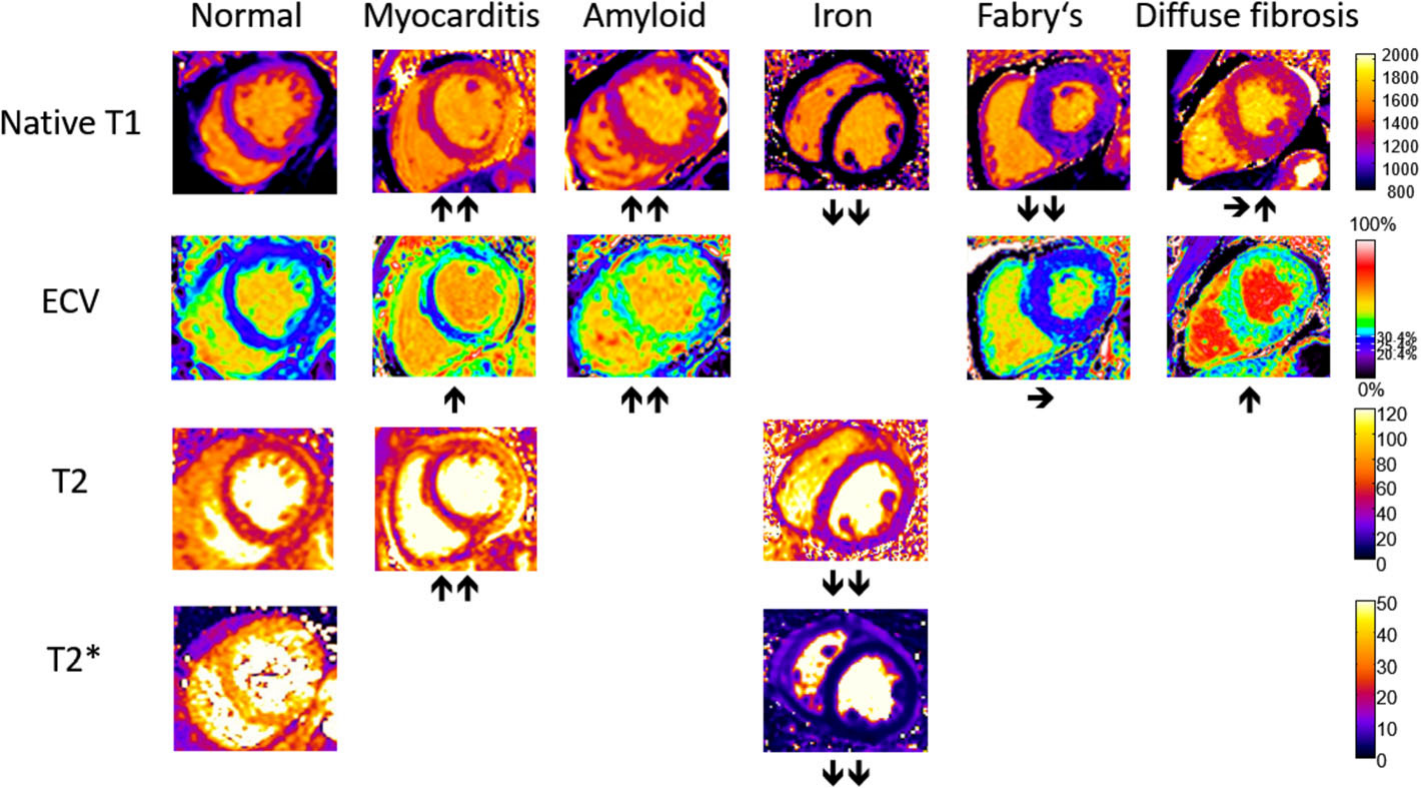
Typical appearance of T1, T2, T2*, and ECV maps in healthy subjects and in patients with myocardial disease. Arrows denote relative change in respective parametric maps. Courtesy of P.K

1. Parametric mapping is useful in patients undergoing evaluation for suspected myocardial disease, and masses.
2. In the clinical scenarios of potential iron overload, amyloidosis, Anderson-Fabry disease, and myocarditis, cardiac mapping provides unique information to guide clinical care and should be applied (Tables 2 and 3; Fig. 1).
3. Parametric mapping should be considered in the diagnostic evaluation of all patients with heart failure and unexplained troponin elevation.
4. The choice of CMR mapping techniques and protocols should be guided by the clinical context.
5. In patients receiving extracellular gadolinium-based contrast agents, routine assessment of ECV may be reasonable.

**Table 2:** Clinical utility of parametric mapping techniques ordered by pathophysiologic mechanism and tissue characteristics. ++ = useful; + = potentially useful;? = unknown; − = not useful. *: Diffuse/global refers to findings affecting the majority of the myocardium, whereas focal/regional refers to localized, including patchy abnormalities

		T1 (native)	ECV	T2	T2*
Infiltration	Iron	+	?	+	++
	Amyloid	++	++	?	–
	Anderson-Fabry	++	–	+	–
Acute myocardial injury	Edema	++	+	++	?
	Necrosis	++	++	+	++
	Hemorrhage	+	?	+	++
Fibrosis	Diffuse/global*	+	++	?	–
	Focal/regional*	+	++	–	–

**Table 3:** Clinical utility of parametric mapping techniques according to expert opinion

Proven clinical utility	Iron deposition
	Amyloid disease
	Anderson-Fabry disease
	Myocarditis
Potential clinical utility	Cardiomyopathy
	Heart failure
	Congenital heart disease
	Acute/chronic myocardial infarction
	Myocardial ischemia
	Suspected transplant rejection
	Athlete’s heart
	(Para-)cardiac masses

Centers undertaking parametric mapping should fulfill the site requirements described in Rationale and diagnostic potential of parametric mapping techniques.

## Recommendations part II: Implementation

### Site preparation and normal values

#### CMR systems

1. T1, ECV and T2 mapping are typically performed at 1.5 or 3 Tesla (T).
2. T2* mapping for iron overload currently should be performed at 1.5 T.

#### Pulse sequence schemes

1. CMR mapping sequences for clinical use should have a published clinical evidence base.
2. Where mapping is clinically reported, the use of a commercial pulse sequence is preferable if supplied by the manufacturer. Other pulse sequences can be considered, but both need to fulfill (1) above.

#### Normal/reference ranges

1. For native T1 and T2 mapping, local results should be benchmarked against published reported ranges, but a local reference range should be primarily used.
2. Reference ranges should be generated from data sets that were acquired, processed, and analyzed in the same way as the intended application, with the upper and lower range of normal defined by the mean plus and minus 2 standard deviations of the normal data, respectively.
3. If local reference ranges are not available for native T1 and T2 mapping, quantitative results should not be reported clinically.
4. The required precision of the local reference range depends on the proposed clinical application:
  - For scenarios with large-magnitude biological changes (e.g. T1 for establishing diagnosis in amyloid, iron, Anderson-Fabry disease, and acute myocardial injury), lower precision is acceptable; e.g. native T1 and T2 reference ranges on the basis of 15 healthy subjects or 20 normal individuals (e.g. referred for CMR without any abnormal findings) may be sufficient.
  - For small-magnitude biological changes (e.g. diffuse myocardial fibrosis), high precision is required for native T1 and T2 mapping; e.g. gender +/− age adjusted reference ranges derived from 50+ healthy subjects.
  - For ECV, reference ranges from the literature using the same CMR system and same pulse sequence may be acceptable, as the dependence of ECV on field strength, sequence choice and imaging parameters appears lower than for native T1.
  - For T2*, a 3-tier risk model (low risk, >20 ms; intermediate risk, 10–20 ms; and high risk, <10 ms) for cardiac iron overload should be used if images are acquired at 1.5 T with ≥8-point gradient echo pulse sequences.
  - Tracking changes over time requires the use of identical imaging parameters or high-precision reference ranges (see b).

#### CMR system-related changes of normal/reference ranges over time

1. Once a reference range is established, the major scan parameters (slice thickness, flip angles etc.), contrast agent/dose and systolic/diastolic phase should not be changed.
2. Regularly repeated phantom-based quality control is recommended to ensure that status and stability of the CMR system have not changed significantly during the time between establishing normative values and clinical scanning.
3. Phantom-based quality control should be performed every time there is a change to the CMR system (hardware, software), a software installation, and every 3 months.

### Imaging protocols

#### General recommendations:

1. Native T1, T2, and T2* are measured in the absence of contrast agents (at least 24 h from the last dose, if any, in patients with normal renal function).
2. Motivation and detailed instructions of patients are important to avoid incomplete breath-holds or motion artifacts.
3. In-plane motion correction is recommended if available, but is not a replacement for breath-holding in non-navigated techniques.
4. Volume-selective B0 shimming focused on the heart is highly recommended at 1.5 T, and essential at 3 T. B1 (radiofrequency) volume shimming is recommended at 3 T.
5. In-plane resolution should not be increased to levels where the resulting acquisition duration of the source images exceeds the time frame within the cardiac cycle where data can be acquired without blurring effects by the giving technique, with consideration of the subject’s heart rate.
6. Diastolic image acquisition is recommended if there is a regular heart rhythm.
7. In patients with tachycardia, specific sequences designed for higher heart rates can be useful.
8. In patients with atrial fibrillation, image acquisition should be repeated to allow for averaging of the results. Systolic readout has been shown to produce robust T1 maps in tachyarrhythmias but requires specific normal values.
9. In patients with pacemakers or implanted cardiodefibillators (ICDs), CMR parametric mapping is not reliable unless specific shimming algorithms or sequences can be used to minimize the impact of artifacts.

#### T1 mapping/ECV

1. Optimized acquisition schemes for post-contrast acquisitions can be used to gain precision.
2. For Look-Locker-based techniques (e.g. MOLLI), correction for readout-induced deflection of T1 relaxation is required (Look-Locker correction).
3. An extracellular contrast agent with non-protein bound distribution should be used for the assessment of ECV.
4. Gadolinium based contrast doses of 0.1 – 0.2 mmol/kg are recommended.
5. Hematocrit for the calculation of ECV should be obtained immediately before the scan if possible, otherwise within 24 h of scanning.
6. For ECV measurements, post-contrast T1 mapping should be performed 10 – 30 min post contrast administration.
7. Split dose protocols (e.g. in adenosine perfusion scans) can be used to assess ECV. The timing should be taken from the last dose.

#### T2 mapping

1. T2-prepared balanced steady-state free precession (bSSFP) or gradient echo pulse sequences with a minimum of 3 source images are recommended.
2. GraSE or turbo spin echo (TSE) approaches may be appropriate if published data on accuracy and precision are available and favorable.
3. Two-parameter fitting is appropriate.
4. Saturation pulse acquisition may help to remove T1 recovery bias.

#### T2* mapping

1. If available, T2* mapping should be performed at 1.5 T (see also Rationale and diagnostic potential of parametric mapping techniques).
2. Multi-echo gradient echo with 8 equally spaced echoes ranging from 2 to 18 ms may be used at 1.5 T.
3. A dark-blood approach is recommended if available.
4. T2* for the assessment of iron overload an interventricular septal region-of-interest (ROI) is recommended
5. For the concomitant assessment of liver T2*, the use of fat saturation and the shortest echo time (TE) available are recommended.

#### Scan planning and acquisition

**Fig. 2:**
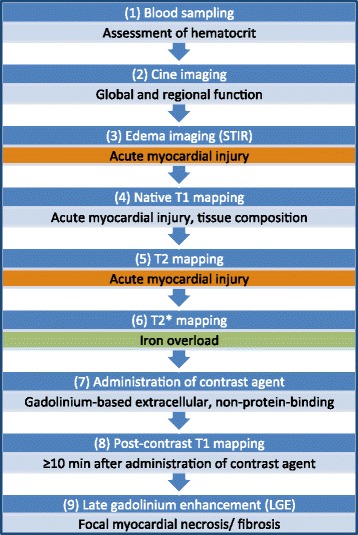
General imaging protocol for myocardial tissue characterization including parametric mapping. The choice of components depends on the clinical scenario (see Tables 2 and 4). For slice orientations see Table 4. STIR = Short TI inversion recovery. 1: Should be obtained immediately before the scan if possible, otherwise within 24 h of scanning. Not necessary if synthetic ECV available. 3: Search tool for focal myocardial edema. Dispensable if high-quality T1 and/or T2 mapping is performed with full LV coverage. 3&5: Not necessary in non-acute disease. 6: Not necessary if iron is not of interest. 7–9: Not necessary if both focal and diffuse myocardial fibrosis are not of interest

1. Added pulse sequences should not compromise the primary study indications.
2. Proper adjustment of main magnetic field shim and center frequency should be assured to minimize off resonance.
3. Through-plane partial volume effects should be minimized where possible by choosing slice orientations that are located orthogonal to the target structures. Caution is required in short axis views of the apex.
4. Image quality should be reviewed during acquisition (e.g. by monitoring sequence sounds and electrocardiographic (ECG) gating), and by looking at source images, error maps, and other quality control maps). Scans should be repeated if sub-optimal or non-diagnostic.
5. Native and post-contrast T1 maps should be acquired using the same slice prescription and other scan parameters at the same cardiac phase (but T1 sampling scheme may be changed – see also T1 mapping/ ECV).
6. A comprehensive imaging protocol for myocardial tissue characterization including parametric mapping is presented in Fig. 2. Disease-specific recommended slices and approaches are given in Table 4. We give the following recommendations:
  a) For global/diffuse disease, a basal and mid short axis map should be acquired with an optional single long axis map.
  b) For patchy disease, the acquisition of at least one long axis map is mandatory (4-chamber for amyloid to visualize base-to-apex gradient, 3-chamber for Anderson-Fabry disease to assess basal inferolateral scar) in addition to basal and mid short axis maps.
  c) For focal and/or acute disease, additional short axis maps should be added and should cover an area of maximal abnormality and an area of apparently minimal abnormality (as determined on cine and/or T2-weighted images) or the whole left ventricle (if information from cine and T2-weighted images should not be available at the time of image acquisition).
  d) Whole heart coverage may add diagnostic yield but risks disproportionately long imaging protocols and patient fatigue.
7. Inline ECV maps (including synthetic, if the relationship between native blood T1 and hematocrit is known for the pulse sequence and field strength) can be a useful alternative to manual ECV calculations.

**Table 4:** Recipe table for specific parametric mapping protocols. SAX = short axis slice, 3Ch = 3 chamber view, 4Ch = 4 chamber view, T1 = T1 mapping, T2 = T2 mapping, T2* = T2* mapping

Scenario	Pulse sequences/slice orientations	Breathholds
Amyloid	T1 mid and basal SAX, 4Ch	**7**
	repeated post contrast	
	T2 mid SAX,	
	T2* -	
Anderson-Fabry	T1 mid and basal SAX, 3Ch	**4 – 7**
	repeated post contrast (research)	
	T2 basal SAX	
	T2* -	
Iron overload	T1 mid and basal SAX, 4Ch	**6**
	not post contrast	
	T2 liver single transverse	
	T2* mid SAX, liver single transverse	
Myocarditis, acute myocardial infarction, other regional disease	T1 SAX multi-slice, long axis (through region of hyper-intensity on STIR or regional wall motion abnormality on cine)	**16 – 25**
	repeated post contrast	
	T2 SAX multi-slice	
	T2* -	
Diffuse fibrosis	T1 mid and basal SAX, 4Ch (research)	**6**
	repeated post contrast	
	T2 (research)	
	T2* -	

### Visualization and analysis

1. Reporting clinicians should learn how to review source images and quality control maps to ensure registration/significant artifacts not present.
2. Maps may be displayed in color if the color look up tables are set according to site-specific ranges of normal, or in gray scale in combination with appropriate image processing, to highlight areas of abnormality.
3. For global assessment and diffuse disease, a single ROI should be drawn in the septum on mid-cavity short-axis maps to avoid lung, liver and veins as sources of susceptibility artifacts.
4. In case of artifacts or non-conclusive results on mid-cavity ROIs, basal ROIs can be used for validation.
5. For focal disease, additional ROIs might be drawn in areas of abnormal appearance on visual inspection. Very small ROIs (<20 pixels) should be avoided.
6. ROIs should be checked if generated automatically.
7. Drawing ROIs on greyscale may avoid bias.
8. Myocardial ROIs should be placed accurately to minimize partial volume effects from adjacent blood pool or extra-myocardial tissues.
9. ROIs should be drawn independently of late gadolinium enhancement (LGE) fibrosis imaging. It is acceptable for ROIs to exclude infarcts (i.e., include remote myocardium) and include non-ischemic LGE.
10. There is currently no specific recommended/preferred analysis software package. The image reader should be trained with the local standards and with the analysis software package of choice and be aware of and familiar with the appearance of artifacts.
11. Sensitivity of mapping techniques to confounders such as heart rate and magnetic field inhomogeneities should be considered during interpretation.

### Reporting

1. For clinical reports, the type of pulse sequence, reference range, and type/dose of gadolinium contrast agent (if applied) should be quoted.
2. Mapping results should include the numerical absolute value, the Z-score (number of standard deviations by which the result differs from the local normal mean; if available), and the normal reference range.
3. An interpretation should be given as normal, mild, moderate, or severe increase/decrease.
4. Best practice is defining the severity of deviation based on prognostic data. If such data are not available (as is the case for most applications of cardiac mapping) and no published suitable scheme is available, the findings may be classified as mild, moderate, and severe, referring to tertiles of the known spectrum of disease severity.

## Rationale and diagnostic potential of parametric mapping techniques

Cardiac mapping techniques extend the diagnostic capabilities of CMR by enabling the quantification of CMR signal changes on an absolute scale. Conventional CMR techniques require a reference tissue (intracardiac: remote myocardium, or extracardiac: skeletal muscle) to detect alterations of myocardial tissue composition. The direct quantification of myocardial tissue properties effectively eliminates the need for such a reference tissue, which makes parametric mapping the first CMR tool that allows for direct assessment of diffuse myocardial disease.

**Fig. 3:**
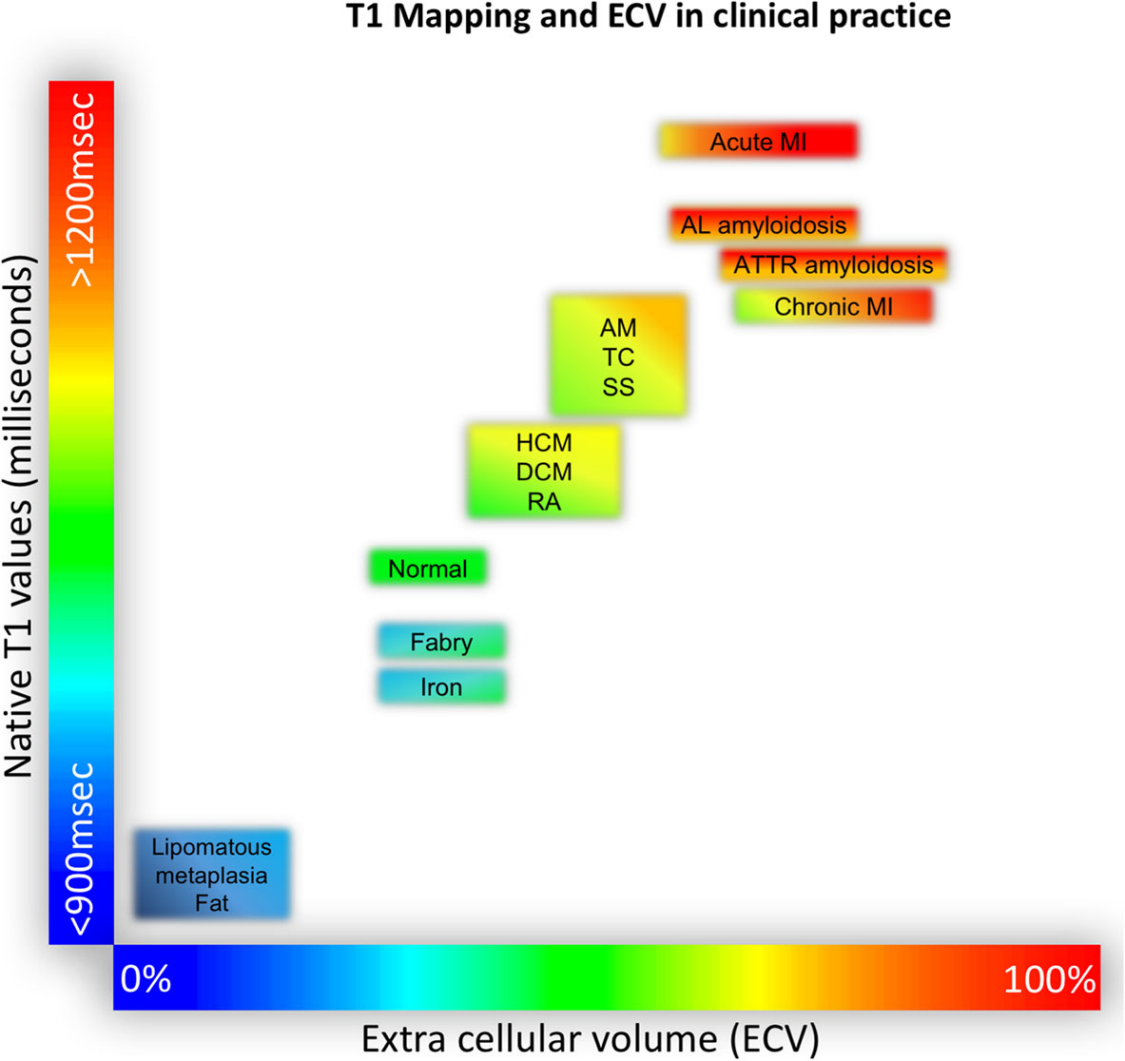
Alterations of T1 and ECV in different myocardial diseases (reproduced with permission from [193]). T1 values refer to MOLLI-based techniques at 1.5 T

The different cardiac mapping techniques provide specific parameters of the myocardium (native T1, T2, T2*, and ECV). While changes of these parameters are not specific for single diseases, they might serve as valuable biomarkers in the context of specific clinical scenarios, as changes of these parameters can be grouped into different patterns depending on the underlying pathology and reflect significant alterations in myocardial tissue composition (Fig. 3), Table 5.

**Table 5:** Typical alterations of T1, T2, T2* relaxation times and ECV according to pathology. For further details see Captur et al. [194]

Measure	Decrease	Mild increase	Moderate or severe increase
Native T1	Anderson-Fabry, iron overload, fat, hemorrhage (athlete’s heart)	diffuse fibrosis, scar, subacute inflammation	amyloid, acute inflammation, acute ischemia, necrosis
ECV	athlete’s heart	diffuse fibrosis	amyloid, necrosis, scar
T2	iron, hemorrhage	subacute inflammation	acute inflammation, acute ischemia, necrosis
T2*	iron, hemorrhage, stress-induced ischemia		

As with any novel diagnostic approach, much of the research initially published in the field of parametric mapping was centered on advances in acquisition methodology. With a multitude of fast and robust mapping techniques described in the literature, some of which are commercially available on modern CMR systems. There is now growing evidence on the clinical value of CMR myocardial mapping from large-scale clinical outcomes trials. In principle, CMR myocardial mapping techniques hold important potential for making diagnosis, risk-stratifying, and monitoring therapy. Thus, parametric mapping can be regarded as a natural extension of comprehensive CMR protocols for the assessment of myocardial disease.

## Available evidence

CMR mapping of T1, T2, T2*, and estimation of ECV in the clinical setting has intensively been studied over the past decade. Until very recently, the necessary pulse sequences and post-processing software have not been commercially available. Scientific developments in the field have mainly been driven by single large CMR centers, while evidence from multi-center trials is limited. Thus, current levels of evidence in the field mainly rely on single-center studies and opinions of experts who perform CMR mapping sequences in clinical routine for several years.

The present recommendations intend to introduce the concept of development steps towards the clinical application of T1, ECV, T2, and T2* mapping. These steps are defined by the available evidence supporting the clinical use of particular mapping sequences for the assessment of distinct disease patterns.

The proposed levels of utility are A) established applicability and usefulness which has been demonstrated by several clinical trials performed by more than one CMR center; B) emerging utility, defined as applications that have been proven useful in only one center; and C) potential applications, i.e. applications that have so far only been tested in experimental settings or animal models.

## Physical and technical background

### Principles of measuring magnetic relaxation times

The most common method for measuring relaxation times is to acquire a series of images in which the time to readout after inversion had been varied over a sufficiently wide range. The relaxation time can then be calculated on a pixel-by-pixel basis by fitting the image intensity of the series against the parameter that was used to vary the relaxation time weight. This pixel-wise relaxation time fitting needs to meet two conditions in order to be accurate and to avoid bias: 1) the variation in weight of other factors (different relaxation times, diffusion, etc.) is negligible or corrected for, and 2) there is negligible physical displacement between the images in the series. In the case of CMR, cardiac and respiratory motion make meeting both conditions more challenging: avoiding motion artifacts limits the pulse sequence choices. Cardiac motion is normally avoided by only acquiring for a sufficiently short duration at mid-systole or end-diastole, while respiratory motion can be avoided or compensated for through breath holding or navigator gating, respectively. Any residual image-to-image displacement (shifts due to respiration or myocardial size differences due to contraction of the heart) can potentially be corrected through affine or non-rigid image registration after the acquisition and before pixel-by-pixel fitting. After the maps have been generated, several segmentation options are available: 1) the entire LV myocardium is segmented to establish an average value or perform a threshold-based analysis to determine areas of abnormality as a percentage of the LV myocardium, 2) the septal region is segmented, 3) the myocardium is divided into 16 segments as defined by the American Heart Association guidelines, or 4) ROIs are drawn in regions of pathology and healthy (remote) tissue for comparison. Option 2 (septal assessment) is mandatory for T2* mapping due to frequent artifacts in other regions of the myocardium (especially off-resonance close to the air-tissue interface), and might also be preferable for the assessment of diffuse myocardial disease by T1 and T2 mapping because of a high level of robustness and ease of omitting contamination from epicardial fat.

### Acquisition and processing strategies

#### T1 mapping and ECV

Several approaches for the quantification of myocardial T_1_ have been described. The majority of sequences utilize multiple single-shot bSSFP acquisitions, using *inversion* preparation (MOLLI [3–5], ShMOLLI [6], ANGIE [7], STONE [8]), *saturation* preparation (AIR [9], SASHA [10], SAP-T1 [11]) or a *combination of the two* (SAPPHIRE [12]).

The modified Look-Locker [13] inversion recovery (MOLLI) sequence is widely used for myocardial T_1_ mapping [3, 5, 14]. MOLLI offers excellent T1 precision, although its T1 assessment is sensitive to magnetization transfer (MT) effects [15], T_2_, flip angle, inversion pulse efficiency, heart rate and off-resonance, which reduces its accuracy [15–18]. Variants of MOLLI, defined by the number of images (or seconds) in a set (i.e. acquired following a given preparation pulse) and the number of recovery heartbeats (or seconds) between sets, have been optimized based on targeted ranges of T1 values and heart rates [16]. The MOLLI 5(3)3 [16] and the Shortened MOLLI (ShMOLLI) variants have emerged as approaches that require reduced scan time at a modest noise penalty [6], with little impact on their practical performance in direct comparison to the original MOLLI scheme [16, 19–22].

Saturation-based approaches typically use a single image acquisition per preparation pulse, minimizing the complex magnetization history of the Look-Locker approaches, and largely eliminating sensitivity to MT, T2, heart rate and off-resonance effects at the price of reduced precision [10, 15, 16]*.* SAturation Pulse Prepared Heart-rate-independent Inversion REcovery (SAPPHIRE) uses a hybrid of saturation and inversion pulses to improve precision of the saturation-recovery approach while maintaining accuracy [12].

Researchers continue to address the challenges of optimizing accuracy and precision with new acquisition and processing methods, for example, with the use of Bloch equation-based T1 corrections for MOLLI acquisitions [23–25] and a free-breathing version of SASHA with improved precision and contrast [26]. Whole heart T1 mapping with 3D ANGIE [7] or Slice-interleaved T1 mapping (STONE) [8] have illustrated full T1 map coverage of the LV with free-breathing acquisitions. In patients with atrial fibrillation, systolic data acquisition might be more robust than diastolic readout, but yields lower T1 values [27, 28].

T1 mapping can be used to estimate the myocardial ECV, a validated surrogate marker of fibrosis [29–31] in the absence of confounders (e.g. infiltration), based on the change in T1 values following the injection of conventional extracellular T1-shortening agents (e.g. Gd-DTPA) [22]. ECV estimation requires repeated T1 mapping acquisitions at baseline (native T1) and post-contrast delivery (typically >10 min post-contrast to approach steady-state conditions). Briefly, the change in 1/T1 (R1) in the tissue and blood pool is used to determine contrast agent concentrations, the ratio of which yields an estimation of ECV, following a correction for red blood cell density in the blood pool (hematocrit). Recently, a simplified approach for the assessment of ECV was presented where the hematocrit was estimated from native values of blood pool T1 (“synthetic ECV”) [32]. Preliminary data suggest that synthetic ECV values correlate with hematocrit-derived ECV, which might allow for abstaining from laboratory tests of hematocrit for the assessment of ECV in the future [33].

For T1 mapping acquisition methods, T1 values are estimated by fitting a T1 recovery curve to each pixel in a series of images with different degrees of T1 recovery using a two- or three-parameter fit [3]. Therefore, motion between images will adversely impact T1 measurements and should be corrected [34, 35]. Methods for the quantification of variability (e.g. quality control maps) in T1 and ECV values in the calculated maps have been described [36].

#### T2 mapping

In the presence of edema, myocardial T2 will increase. T2 weighted CMR imaging is commonly used to assess myocardial inflammation. However, image quality, reproducibility and subjective assessment of T2 weighted images have been limiting factors in its clinical adoption. To overcome these challenges, regional myocardial T2 mapping has emerged to directly quantify local myocardial inflammation and edema. Few studies have investigated the value of T2 mapping in other myocardial diseases [37]. T2 mapping is generally performed by pixel-wise fitting for a T2 decay curve of a series of T2-weighted images. These source images can be acquired by a TSE sequence with varying echo time [38], a bSSFP or spoiled gradient echo (GRE) sequence with an initial T2 preparation module [39–42], or a sequence scheme that combines spin echo excitation with gradient echo readout (GraSE) [43]. In these T2 mapping sequences, images are acquired with different echo times (e.g. 0, 25, and 50 ms) [39] and are used to estimate the T2 values using a two-parameter or three-parameter fit model [44]. Despite the potential of T2 mapping to replace T2-weighted imaging for assessment of inflammation and edema due to its quantitative nature and higher robustness, confounders such as sensitivity to T1 and off-resonance effects have to be considered. Recent studies have thus focused on quantifying the reproducibility [45–50], robustness against artifacts [51], and the influence of motion correction [45, 52]. At the same time, several challenges of myocardial T2 mapping have been overcome by proposing faster image acquisition, reducing bias [44], and increasing spatial coverage [53–55].

#### T2* mapping

T2* relaxation mapping is usually based on GRE sequences and forms the basis for numerous CMR applications [56]. In particular, T2* quantification is currently the method of choice for myocardial tissue iron assessment [57]. In this scenario, images are typically acquired at 8 different echo times ranging from 2 to 18 ms. For cardiac imaging, a segmented GRE sequence combined with ECG gating allows all echo images to be acquired in a single breath-hold. Currently, both a bright blood [58] and a black blood [59] technique are validated and widely used clinically [60, 61]. For the bright-blood technique, images are acquired immediately after the R-wave to reduce artifact because of blood flow and myocardial wall motion. For the black-blood technique, a double inversion recovery pulse is used to null the signal from blood, and data acquisition is extended to late diastole with minimal cardiac motion. In comparison, the black-blood technique produces less bias and reduced inter-observer variability and is hence recommended if available [62].

For the measurement of myocardial T2* in vivo, a mid-ventricular short axis slice is acquired and a homogeneous ROI is defined encompassing both sub-epicardial and sub-endocardial regions, as iron is preferentially stored in the sub-epicardial compared with the sub- endocardial layers [62]. *The analysis is restricted to the septum* to reduce susceptibility artifacts. A multi-slice T2* approach has also been proposed, which may have advantages for the evaluation of myocardial hemorrhage in acute myocardial infarction by providing whole-heart coverage. The single-slice technique based on the septal analysis remains accepted practice [63].

## Quality control

Changes in CMR system hardware and software may lead to changes in mapping sequence parameters (see Normal values). During image acquisition, mis-triggered heart beats, motion (cardiac and respiratory), and off-resonance or other artifacts may lead to inaccurate measured relaxation times (see [64], electronic supplementary material). When possible, breath-held acquisitions with data collection during the quiescent period the cardiac cycle, typically mid-diastole, is preferred. Pixel-by-pixel parametric maps still benefit from breath-holds, even if motion-correction algorithms are used, as motion correction algorithms can only correct in-plane but not through-plane motion. These algorithms can incorrectly co-register anatomy, and corrupt image quality and accuracy [48], so it is imperative to view the motion-corrected raw images to ensure that the motion correction had worked properly. The operator should ensure that there are no mis-triggered or skipped heart beats during data acquisition. Once acquired, the user should immediately inspect the raw images, quality control maps (e.g. R^2^ or error), if available, and the parametric maps to assess for quality, reacquiring images as necessary. For instance, motion may manifest as obvious diaphragmatic motion or variation in cardiac phases on the raw images, areas of poor curve fit on quality control maps, or blurring of tissue borders on T1/T2 maps. For T2* measurements, poor breath holding and cardiac motion typically manifests as ghosting, and may significantly affect image and data quality. Off-resonance artifacts may be particularly prominent at 3 T, and the use of a local box shim is essential to reduce off-resonance variation across the heart.

## Normal values

In healthy subjects, myocardial tissue exhibits a very uniform composition and thus possesses very regular magnetic properties. As a result, native T1, T2, and T2* values from normal myocardium are highly reproducible and show relatively narrow ranges when acquired under the same conditions.

In practice, the term “same conditions” becomes highly relevant. If these conditions change, it becomes impossible to judge if changes in tissue parameters results are due to a pathologic state or simply to a change in the way the measurement was performed.

The conditions for parametric mapping can be divided into two areas:

1. *Physical/biological confounders.* These include magnetic field strength and temperature, meaning that native T1, T2, and T2* values are not directly comparable between 1.5 T and 3 T, T1 will increase by approximately 1% for every 1 °C increase in body temperature [65], and T2 shortens with increasing temperature [66]. Temperature dependency is usually neglected as extremes of body temperature (pyrexia, hypothermia) are not typical features of patients undergoing a CMR scan, but nonetheless should be considered in the unusual event that a febrile (or hypothermic) patient undergoes CMR parametric mapping.
2. *Methodological confounders.* The acquisition and processing of parametric maps require several technical steps. There are multiple technical options for each of these steps, each of those introducing different types and degrees of error to the measurement. For example, the use of inversion recovery Look-Locker based acquisition schemes for T1 mapping introduces a significant negative offset to T1 values as compared to saturation recovery sequences; the use of insufficient waiting time for signal recovery in inversion recovery T1 methods and T2-prepared mapping can introduce heart rate dependence; and low flip angles can lead to low SNR in all approaches.

This problem is complicated by the fact that there is no reference technique to determine the “real” myocardial relaxation times in-vivo, as in-vitro reference techniques cannot be applied to live human hearts and hearts from other species (that might be subjected to several hours of scanning) do not exhibit the exact same normativevalues as human hearts.

As a consequence, native parameter values should only be compared to other parameter values if they are obtained under similar conditions. In other words, the acquisition scheme, field strength and processing approach should be the same, and the results should be reported along with corresponding reference ranges for the given methodology (see **Reporting**). This situation actually resembles that of other biomarkers including serologic tests, where reference values of the local laboratory are usually provided along with the results sheet. In the future, it might become possible to normalize native parameter results to a “standard parameter” based on phantom or software calibration methods [67].

Apart from these variations caused by confounders, there are subtle differences in myocardial T1 and T2 (but not T2* [68]) that are related to gender and (less so) age. In 1231 participants of the Multi-Ethnic Study of Atherosclerosis (MESA) aged 54 to 93 years, women showed stable native T1 throughout different age groups whereas men had lower native T1 at 54 to 63 years that increased in higher age groups and approached that of women in the group of >84 years [69]. In a study of 342 healthy subjects, native T1 values of women were higher up to the age of 45 years [70]. Similarly, a study in 74 healthy subjects revealed higher myocardial T2 in women and in subjects with greater age [47, 71]. Further insights are expected from on-going other population-based studies [72]. Taken together, the magnitude of gender- and age-related differences for myocardial relaxation times in these studies reached approximately 0.5 standard deviations from the normal mean. While these effects seem negligible in diseases with alterations of high magnitude (e.g. amyloidosis), they become relevant if pathologic processes with more subtle impact on native relaxation times such as diffuse myocardial fibrosis are of interest. As a consequence, non-specific normal ranges might be appropriate for high-magnitude disease states, while the assessment of low-magnitude pathologies requires the use of granular normal ranges according to gender and to a lesser extent age, which are currently not available for all acquisition approaches.

## Clinical applications: Current state

### Mechanisms of myocardial injury

T1, T2, and T2* times of a given pixel in a respective parametric map represent a composite signal of the corresponding tissue voxel. Changes of myocardial tissue composition lead to alterations of T1, T2, and T2* times. Such changes usually occur in the same direction (i.e. shorten or lengthen) for all three magnetic properties but might not reach the same degree in all of them, depending on the underlying process. ECV behaves different in that it only reflects alterations of the extracellular component of the myocardium. Table 10 gives an overview on principal directions and degrees of changes in parameters depending on pathology.

### Acute myocardial disease

#### Acute ischemic injury

As acute myocardial edema develops in areas of acute ischemia and infarction, native T1 and T2 relaxation times prolong, whereas post-contrast T1 time shortens, compared to remote myocardium [14, 73, 74]. Both native T1 and T2 mapping correlate well with the area-at-risk measured by microspheres in animal studies [75], and may be used to delineate the area-at-risk and determine myocardial salvage in clinical applications [76, 77]. With the development of significant interstitial edema and other changes in the vasculature, ECV is expanded in the areas of acute myocardial injury and infarction [78]. Compared to T2-weighted imaging, native T1 mapping was superior in detecting areas of injury in non-ST elevation myocardial infarction (NSTEMI), and similar in STEMI [79]. Furthermore, native T1 and T2 mapping may provide prognostic information through the identification of the infarct core [80] and intra-myocardial hemorrhage [81], both of which are associated with an adverse prognosis. Post-contrast T1 and ECV changes have been identified in the remote myocardium early in the course of acute infarction [82–84], suggesting that adverse cardiac remodeling may commence at the time of infarction and is not simply a consequence of longer term hemodynamic stress. T2 mapping may be used to track the resolution of myocardial edema, and T2* imaging may be used to assess for intramyocardial hemorrhage and reperfusion injury post infarction [85].

#### Acute inflammation

Increases in myocardial free water content, as occurs in acute myocardial edema and inflammation, prolong T1 and T2 relaxation times [73, 86]; where myocardial edema is extra-cellular, this will also expand the interstitial space and, hence, ECV. Both T1 and T2 mapping are sensitive to detecting acute myocardial edema and inflammation in animal models [73, 75, 86] and in clinical patients who present with various forms of acute myocardial injury, including myocardial infarction, stress-induced (a.k.a. Tako-tsubo) cardiomyopathy and myocarditis [64, 79, 87–89]. Mapping techniques possess a number of technical advantages over conventional T2-weighted imaging for detecting myocardial edema and inflammation, and have demonstrated superior diagnostic performance in the clinical setting [64, 79, 87, 89]. T1, ECV and T2 mapping have clinical utility in the diagnosis of acute myocarditis, shown by a number of clinical studies, and may be used in conjunction with the Lake Louise Criteria [90, 91]. Additionally, mapping techniques are sensitive to less acute presentations of inflammation, and are able to detect subclinical forms of myocarditis as part of systematic inflammatory diseases, such as rheumatoid arthritis, lupus erythematosus, systemic sclerosis, pheochromocytoma, human immunodeficiency virus infection and cardiac sarcoidosis [92–97]. Mapping techniques may also have emerging roles in the diagnosis of cardiac transplant rejection [98], and differentiation of athlete’s heart from dilated cardiomyopathy, which may have an inflammatory component detectable using parametric maps [99].

### Heart failure with reduced or preserved ejection fraction

#### Aortic stenosis

While the key measure determining the need for valve replacement in aortic stenosis is the degree of valvular obstruction, the myocardium also undergoes progressive changes that can lead to deteriorating cardiac performance and increased morbidity and mortality [100]. The degree of LV hypertrophy in aortic stenosis is independently associated with a higher rate of cardiovascular events [101], and recent T1 mapping data suggest that myocardial ECV is a stronger predictor of adverse cardiovascular outcomes than the extent of LV hypertrophy. A number of T1 mapping techniques have already been shown to correlate with the degree of histological interstitial fibrosis in aortic stenosis patients [102, 103]. Compared to healthy controls, patients with aortic stenosis have a higher ECV [30], suggesting a maladaptive response to pressure overload. In these patients severe diastolic dysfunction is associated with higher ECV levels, implying a mechanistic link between interstitial fibrosis and myocardial stiffness. The notion that pathological LV remodeling in aortic stenosis is at least in part driven by increasing levels of interstitial fibrosis suggests that in the future T1 mapping may play a role in predicting future cardiovascular outcomes in aortic stenosis, or even help to time intervention. Although the principle determinant of regression of hypertrophy following aortic valve replacement for severe aortic stenosis may be a reduction in cell volume rather than regression of interstitial fibrosis [30], studies are currently underway aimed at assessing the role of ECV measurement in the selection of patients for aortic valve replacement. Furthermore, it has been suggested that coronary vasodilation may also contribute to increased native T1 via intravascular volume expansion in severe aortic stenosis [104].

#### Arterial hypertension

Similar to the response to pressure overload in aortic stenosis, CMR T1 mapping studies in patients with arterial hypertension have demonstrated small degrees of expansion in ECV that parallels the development of LV hypertrophy [105]. Furthermore, the development of ECV expansion as a consequence of hypertension is associated with reduced cardiac performance, suggesting a mechanism by which hypertrophy in hypertension may evolve into a more malignant phenotype [106, 107]. In addition, T1 mapping may play a diagnostic role in discriminating patients with LV hypertrophy due to hypertrophic cardiomyopathy from those with hypertrophy secondary to arterial hypertension [108]. This distinction is not merely academic, as hypertension is a common finding in the community and there are important differences in the management of patients with hypertrophic cardiomyopathy compared to those with hypertensive heart disease. Finally, of great interest would be the demonstration of regression of interstitial fibrosis following antihypertensive therapy. In one study, following marked blood pressure reduction in a cohort of hypertensive patients who underwent renal denervation, there was a significant reduction in the blood-tissue partition coefficient, lambda (a surrogate of ECV), in those who underwent renal denervation versus controls [109].

#### Amyloid disease

Two types of amyloidosis commonly infiltrate myocardium: immunoglobulin light-chain derived (AL) and transthyretin (ATTR) amyloid. While these two types have different natural history and prognosis, cardiac involvement drives outcome and therapeutic choices in both. Early recognition and therapy are critical in AL amyloidosis when cardiac involvement is detected. CMR with LGE has been shown to be a valuable tool in cases with typical subendocardial tramline pattern, which is replaced by transmural RV and LV enhancement at later stages. However, atypical patterns have been described and renal impairment restricting contrast use is common in amyloid. Native T1 mapping may allow for making the diagnosis of cardiac amyloidosis without the need for gadolinium contrast application, although the normal T1 in renal disease is not yet well defined [110–112]. ECV appears to be a surrogate marker for the amyloid burden and carries prognostic value [113]. In early disease, native T1 and ECV are elevated before LGE appears, although these changes are initially non-specific and thus only clinically useful when the pre-test probability is high. Once subendocardial LGE appears, ECV elevation in remote areas begins to be diagnostic, as diffuse fibrosis rarely increases the ECV above 40%. ECVs of >55% appears in transmural LGE. The T1 and ECV are subtly divergent in established AL vs. ATTR with a higher ECV in ATTR, a higher native T1 in AL, and greater cell volume expansion in ATTR [114]. Particularly in the elderly, occult ATTR amyloid confounds other diseases such as heart failure, hypertrophic cardiomyopathy, and aortic stenosis, although bone tracer scanning appears more sensitive [115]. ECV can be used to track therapy in amyloid [116], and might be able to track amyloid regression.

#### Anderson-Fabry disease

Anderson-Fabry disease is a rare X-linked lysosomal storage disease that causes LV hypertrophy and eventually fibrosis and heart failure. The underlying pathology is intra-cellular accumulation of glycosphingolipid. Native T1 in this disease is low, unlike any other cause of hypertrophy except for iron overload (see **Iron overload**), in around 85% of all subjects with LV hypertrophy, though to be directly related to sphingolipid storage [117, 118]. This is highly characteristic and diagnostic, despite the rarity of Anderson-Fabry and consequent low pre-test probability. T1 is low in around half of patients with Anderson-Fabry even in the absence of hypertrophy, making T1 mapping a useful test for early cardiac involvement and raising the possibility of early therapy to prevent the downstream events and overt disease.

Patients with Anderson-Fabry frequently show a characteristic mid-wall LGE pattern in the basal inferolateral wall, which often appears thin. Recent data suggest that in this area, in contrast to LGE from other origin, there is an increased native T1 and T2 even in the absence of wall thinning, with the T2 elevation corresponding to elevated levels of blood troponin. This suggests that the lesions on LGE may reflect chronic, active inflammation [119]. This important finding may be a critical link of underlying mutation to a final fibrotic phenotype and may point to a pathway common to other non-ischemic LGE findings and disease development.

#### Iron overload

Cardiac iron overload is a serious condition, caused either by repeated blood transfusions for anemia (e.g. in thalassemia major) or increased intestinal iron absorption (e.g. hereditary hemochromatosis). Iron overload leads to severe heart failure and lethal arrhythmias but can be treated effectively if diagnosed early. T2* mapping is an accurate and reliable method for the quantification of cardiac iron load [120]. It is reproducible across different CMR systems [121, 122], and identifies patients at risk for heart failure or arrhythmia from myocardial siderosis [123]. Furthermore, T2* mapping can be used to monitor disease progression and therapy [124–126], and currently is the only parametric mapping technique that is recommended in disease-specific clinical guidelines [62].

Recently, T1 mapping has also been evaluated for the assessment of cardiac siderosis, and compared with T2 and T2* in a substantial population with and without cardiac iron overload [127], showing considerable scatter between techniques. In patients with only mild increases of cardiac iron, non-contrast T1 mapping showed superior reproducibility as compared to T2* measurements [128], but there might be little relevance of this finding in significant iron overload states causing heart failure.

ECV may be increased in patients with thalassemia major and is associated with cardiac iron overload [129]. However, ECV correlated significantly with lowest historical T2* measurements but not with systolic function. Thus it is currently unknown whether ECV has a role in the management of cardiac siderosis patients.

#### Diffuse myocardial fibrosis and cardiac remodeling

Myocardial fibrosis occurs in a continuum from mild to severe where excess collagen (concentration) appears in the myocardial interstitium [130]. Accordingly myocardial native T1 and ECV increase, whereas post contrast T1 decreases [2]. ECV is well suited to measure interstitial expansion occurring with fibrosis (or amyloidosis) with extracellular gadolinium-based contrast agents. ECV simply *quantifies* the interstitial presence of gadolinium relative to the plasma. Accordingly, histologic validation data overall show best agreement with ECV compared to other T1 metrics based on R2 values (i.e., the proportion of variation in a variable explained by another variable) [131, 132]. Despite the potential confounding introduced by capillary rarefaction or myocardial edema (since myocardial gadolinium presence includes the myocardial vasculature), most validation studies report high R2 values ≥0.6 [29, 103, 133–138]. The intra-myocardial vascular compartment is another potential confounder in the setting of vasodilation (e.g., adenosine [104, 139, 140]), so the clinical context for ECV measures must be known for optimal interpretation. Since LGE detection of fibrosis depends on its spatial heterogeneity, LGE is not designed for quantifying fibrosis in noninfarcted myocardium and is not validated as a quantitative metric for this purpose. Nonetheless, LGE can often identify cardiac amyloidosis (see above) [113]; thus LGE can assist discrimination of the cause of elevated ECV, i.e., myocardial fibrosis versus cardiac amyloidosis.

ECV dichotomizes the myocardium into its primarily cellular compartment and predominantly interstitial compartment (including the myocardial vasculature) [2]. While myocardial fibrosis may follow myocyte loss due to various injuries, it also may occur with primary fibroblast activation. The *positive* correlation between myocardial fibrosis (whether by ECV or histology) and LV mass suggests significant primary fibroblast activation since myocyte loss would decrease LV mass [132]. This information is relevant when appraising potential therapeutic targets.

Emerging data reveal that many cardiac insults culminate in myocardial fibrosis, and the extent of fibrosis can vary across disease categories [78]. The extent of myocardial fibrosis regardless of cause or disease category then appears to govern vulnerability to adverse outcomes (death or heart failure) [141]. ECV appears to reflect the extent of myocardial fibrosis and has been validated against collagen volume fraction [29, 103, 133–138]. ECV has been shown to be reproducible [136, 142–147], predict outcomes [148–155], and provide “added prognostic value” manifest by reclassification metrics [141]. Thus, ECV quantification of interstitial expansion remains a powerful tool to investigate myocardial remodeling, especially when combined with ancillary clinical data.

#### Primary cardiomyopathy

Dilated cardiomyopathy and hypertrophic cardiomyopathy are associated with the development of diffuse myocardial fibrosis. In both groups of patients, native T1 was found to be increased not only in areas corresponding to LGE but also in areas without LGE, hence suggesting that native T1 can detect areas of tissue pathology beyond those detected by LGE [156]. In patients with dilated cardiomyopathy, high native myocardial T1 is associated with an increased risk for cardiovascular events and heart failure [157]. Furthermore it could be shown for both dilated cardiomyopathy [158] and hypertrophic cardiomyopathy [159] that ECV is increased not only in patients with typical phenotype, but also in asymptomatic relatives without clinical findings but with positive phenotype. Whether ECV provides additional information in these patients for the prediction of ventricular arrhythmia is under investigation. There are no data on the diagnostic value of parametric mapping in patients with arrhythmogenic right ventricular cardiomyopathy (ARVC). The fibrofatty replacement, which is a typical finding in this disease, should theoretically lead to alterations of both native T1 and ECV. However, the thin RV free wall prevents the application of breathhold parametric mapping techniques, which do not provide high-enough spatial resolution. Respiratory-gated segmented techniques might circumvent this problem in the future [7].

#### Valvular disease

Fibrotic remodeling as a consequence of chronically increased afterload has long been recognized. Even young patients with aortic stenosis exhibit increased ECV, which correlates with the degree of diastolic dysfunction [160]. In addition to the cardiac remodeling driven by pressure overload, volume overload driven by valvular regurgitation is also associated with adverse cardiac remodeling. In contrast to stenotic valvular lesions, most patients with valvular regurgitation develop significant cardiac remodeling prior to the development of symptoms; therefore the role of CMR in characterizing subclinical myocardial changes is of particular importance. In patients with chronic aortic regurgitation, shorter post-contrast T1 time, consistent with diffuse myocardial fibrosis, is present in myocardial segments with impaired function [161], again suggesting a relationship between cardiac dysfunction and interstitial fibrosis. More recently, similar findings have been demonstrated with ECV in patients with mitral regurgitation [162], where changes in ECV were linked with early cardiac remodeling and reduced cardiovascular performance. Taken together these studies suggest a potential future role for CMR T1 mapping in the identification of subclinical myocardial changes due to volume overload, which may help guide future interventional strategies for regurgitant valve lesions.

#### Ischemic heart disease

Besides the severe alterations of native T1 seen in the acute stage (**Acute ischemic injury**), myocardial infarction also causes changes of native T1 in the chronic stages of the disease. However, the heterogeneous nature of these changes both within and in between subjects limit the sensitivity of native T1 for the detection and – even more so – the quantification of the extent of chronic myocardial infarction in a given individual [14, 163]. An increase of native T1 has also been reported for remote areas of hearts with acute myocardial infarcts (see **Acute ischemic injury**), and was associated with LV remodeling and adverse cardiac events [164]. Recently, characteristic patterns of pathologic T1 response to adenosine stress have been demonstrated in patients with significant coronary artery stenosis using native T1 mapping [139, 140]. Apart from direct effects of impaired myocardial perfusion on T1, additional mechanisms that might contribute to these patterns include blood oxygenation level dependent (BOLD) and arterial spin labelling (ASL) effects [165]. Further studies are necessary to answer the question if native T1 mapping can be used as a non-contrast stress perfusion test for the assessment of myocardial ischemia.

### Congenital heart disease

In congenital heart diseases, volume and/or pressure overload are important factors in alterations of myocardial structure and function [166]. In adolescents and adults with Tetralogy of Fallot, for example, the presence of volume overload due to pulmonary regurgitation is associated with expansion of ECV, which in turn is associated with a higher incidence of cardiac arrhythmia [167]. Although Tetralogy of Fallot is a “right heart disease”, LV ECV is elevated as well [168, 169]. Pediatric Tetralogy of Fallot patients from a contemporary era enjoy an overall better myocardial health than previous surgical generations, but an association of both ECV and native T1 in the LV with exposure to cardiopulmonary bypass more than 10 years earlier remains a concern and highlights the need for improved cardioprotection [168]. Furthermore, chronic hypoxemia and genetic disposition have been associated with adverse myocardial remodeling in repaired and unrepaired malformations of the heart. Nearly all CMR T1 mapping studies in Tetralogy of Fallot have demonstrated higher ECV and/or native T1 values in females [167]. This finding together with worse RV function and exercise tolerance [170] suggests that we may need to monitor and treat females differently from males after Tetralogy of Fallot repair.

Patients with physiologically uni-ventricular hearts (so called “single ventricles”) are at particular risk for developing ventricular dysfunction, especially when the dominant ventricle is of RV morphology, as in hypoplastic left heart syndrome [171]. Even at a young age, these patients have been demonstrated to develop elevated ECV, which is associated with reduced myocardial contractility. So far, the prognostic significance of this finding is not known.

Drug trials for the treatment of heart failure in congenital heart disease have been nearly unequivocally disappointing. Information on myocardial health from T1 mapping might potentially allow for a more personalized pharmacological approach. As pharmacologic heart failure therapies have been less effective in these situations than in acquired heart disease, interventional and surgical approaches play a major role. T1 and ECV mapping have been proposed as tools to guide decision-making and timing of such procedures in the course of the disease. For example, the goal of pulmonary valve replacement in post-repair patients with Tetralogy of Fallot may become preservation of myocardial health over and above restoring RV volume, which is currently at the center of timing for pulmonary valve replacement. Thus, longitudinal RV ECV assessments may prove helpful in making the decision when to restore pulmonary valve competency. However, RV disease is hard to assess with common breath-hold T1 mapping approaches as their spatial resolution approaches the low thickness of the RV wall. Even in patients with right ventricles that support the systemic circulation, the RV free wall has been deemed not measurable by T1 mapping [172]. This problem of insufficient spatial resolution is further aggravated in pediatric patients with small hearts. Therefore, while initial studies could demonstrate the diagnostic potential of T1 and ECV mapping in patients with RV disease, meaningful clinical applications seem to warrant the availability of high-resolution mapping techniques, which are based on non-breath hold (navigated) segmented acquisition strategies, examples of which are currently still restricted to research applications (ANGIE [7], SALLI [173]).

Information on the prognostic significance of parametric mapping in pediatric cardiomyopathies and myocarditis is scarce, and it remains unclear whether the experience in adults can be extrapolated to children. In patients with chronic Kawasaki disease, ECV is elevated, including in LGE-negative myocardial segments. It is highest in segments supplied by severely aneurysmal and/or obstructed coronary arteries and is associated with decreased myocardial blood flow and strain. Therefore, CMR T1 mapping may present an opportunity to improve risk stratification and monitoring in Kawasaki disease beyond coronary artery angiography and stress testing. In boys with Duchenne muscular dystrophy, heart failure treatment is typically started or intensified when myocardial dysfunction and/or LGE develop. However, even patients with normal LV ejection fraction and no LGE have elevated ECV and T1 values [174]. This observation suggests that T1 mapping, and perhaps native T1 more than ECV [175], may identify the onset of fibrotic remodeling earlier than LGE and ejection fraction, providing an opportunity for a more timely intervention.

### (Para-)cardiac masses

Mapping techniques may be used for characterizing extra-myocardial tissues, such as masses, pericardial effusion and fat, although current clinical evidence is sparse for these applications. For instance, cardiac masses with high fat content, such as lipomatous hypertrophy of the inter-atrial septum, may be readily apparent on T1 maps [176], demonstrating homogenous and characteristically low T1 values. Simple cysts and pericardial effusion, composed almost entirely of water, are expected to exhibit very long T1 and T2 relaxation times. Cystic lesions without communication with the systemic circulation and its vasculature will show unchanged T1 values post gadolinium administration. Masses that have a high water content and substantial vascularization (and therefore blood volume) also show long T1 and T2 relaxation times, and will have evidence of gadolinium contrast uptake (e.g. low T1 values post contrast) [177]. Due to bias caused by intramyocardial fat seen in inversion- and saturation-recovery T1-mapping techniques, a wide range of native T1 values may be seen in tissues with fat content when a voxel is only partially occupied by fat [178, 179]. It is also important to select mapping techniques that have been validated across a wide range of T1 and T2 values that include the ranges typically seen in the tissues types under study (e.g. cysts with very high T1 and T2 values), to avoid potential misdiagnosis due to underestimation of long relaxation times (e.g. cyst versus malignant tumor). More research and validation against tissue pathology are needed in this area before routine clinical applications for diagnoses.

## Knowledge gaps and areas for ongoing research

### Relaxometry assumptions

The measurement of T1, T2, and T2* relaxation time constants assumes a mono-exponential behavior on a macroscopic scale. This simplifying assumption has proven to be useful in clinical practice for differentiation of tissues and assessment of their state. However, the molecular composition of biological tissue is frequently more complex, leading to multiple compartments with chemical exchange (magnetization transfer) and finite diffusion distances. How the simplified model is affected by more complex molecular composition of tissue and pathology is unclear, and the clinical consequences of the simplified view needs to be studied for various scenarios.

### Confounding factors

The measurement of a parameter of interest such as T1 may depend on other variables such as T2 or patient’s heart rate (HR), and numerous other confounding factors. From a clinical perspective, the consequences of such bias are not fully understood. There are instances for which the confounding factor may make a disease more detectable. However, the interpretation of the shift from baseline normal is no longer clear. For example, an elevated T1 might be due to fibrosis, or might be due to confounding effect of elevated T2 arising from edema. In another example, a decrease in T1 might be due to increased iron concentration, or might be due to off-resonance in the scanner center frequency. Some of the confounding variables are due to patient physiology, but others may be due to scanner adjustments or field inhomogeneities. The extent to which the desired measurement is confounded will depend on both the sequence design and specific protocol. Some of the confounding effects may be disentangled by multi-parametric measurements, or by calibrations scans, but these come at the expense of time and complexity.

### Partial volume effects

Partial volume effects arise from a) the contamination of the desired signal by adjacent tissue such as blood pool or fat, or b) mixtures of tissue within the voxel of interest such as intramyocardial fat. Use of blood or fat suppression may help mitigate partial volume effects but may also affect the measurement of interest. The degree to which partial volume errors influence the measurement depends on aspects such as wall thickness or angulation of the slice prescription.

Myocardial tissue may be heterogeneous with variation on the scale of the voxel resolution. In this instance, the quantitative measurements are smoothed and represent an average, which may not accurately reflect the focal elevation or baseline normal values. For example, the focal elevation of T1 or T2 may depend on the slice thickness when the focal abnormality is thinner than the slice. This is compounded when ROIs are drawn which introduced further averaging. Thus the degree of heterogeneity and size of focal abnormalities is a factor influencing the quantitative measurement and depends on resolution and manner of measurement and reporting. For example, in assessment of iron overload, where the concentration of iron is heterogeneous, it is unknown whether there is clinical significance of the peak concentration and distribution in addition to the average value quantified in typical analysis. The influence of these effects on clinical assessment needs to be characterized.

### Post-processing

The impact of the post-processing methodology (i.e. derivation of relaxation times from a set of source images) on the quality of parametric results can equal or sometimes even exceed that of the acquisition strategy. Consequently, advanced post-processing strategies have been investigated. For T1 mapping, several methods have been proposed that aim to optimize T1 estimation from MOLLI source images [24, 25]. In T2* mapping, there is evidence that a commonly used offset model is fundamentally incorrect [180]. The truncation model is challenging in the presence of severe iron overload [181]. Researchers continue to address this challenge with novel methods. For example, an improved truncation model was extended to black-blood T2* mapping [182] and a noise-corrected mode was developed [183] to cope with both pixel-wise and region-of-interest (ROI)-based curve-fitting. Further studies are needed to identify the methods that provide the best diagnostic accuracy in clinical applications.

### Map analysis

While the assessment of average parameter values of septal ROIs is regarded as appropriate for diffuse myocardial disease, conditions with patchy presentation such as myocarditis (which in some cases changes its distribution from focal to diffuse throughout the course of disease) might require more detailed analysis of regional behavior. Histogram analysis [6] and statistical analysis of tissue heterogeneity [184] have been proposed as means to detect and quantify different tissue populations within ROIs, and might be helpful in the analysis of inhomogeneous myocardial disease.

## Future directions

Future developments in cardiac mapping will likely focus on standardization of data acquisition and post-processing, as well as on optimizing workflows. In parallel, the clinical utility of mapping will have to be carefully explored for various cardiovascular diseases to further define where mapping parameters can firmly establish diagnosis, guide therapeutic decisions, and predict prognosis. The robustness of mapping protocols and results will play an important role in its acceptance for clinical decision-making. Challenges include proprietary approaches of CMR system manufacturers, cost for software packages and the need for calibration of hardware and sequence settings.

On the technical side, accelerated image acquisition such as compressed-sensing techniques [185, 186] may allow for a significant shortening of scan times or for improving image quality or spatial resolution. Three-dimensional (3D) mapping may allow for more complete coverage of the heart and a better characterization of complex regional distribution patterns of disease processes [53, 54, 187].

The combination of mapping with quantitative data from functional (cine) studies can be sufficient to differentiate diseases from physiologic adaptation of cardiac mass, shape and function such as in athlete’s heart [99, 188].

As native T1 and T2 are sensitive to increased myocardial water content and myocardial blood volume, there is current interest to investigate the ability of vasodilatory stress T1 and T2 mapping to detect ischemia without the need for exogenous contrast agents as a novel application of mapping techniques [139, 140].

Several techniques have also appeared that map multiple relaxation times simultaneously (MR fingerprinting) [189–191], with the double advantage of saving time and removing confounders introduced by the interaction of relaxation times. Although the precision, bias, and reproducibility of such techniques need to be carefully quantified, these new techniques augment the available arsenal for various challenging imaging conditions, such as those found in patients with arrhythmia, patchy disease patterns or the inability to perform breath holds.

Non-contrast protocols will be useful for patients with a need for repeated CMR or with kidney failure.

**Fig. 4:**
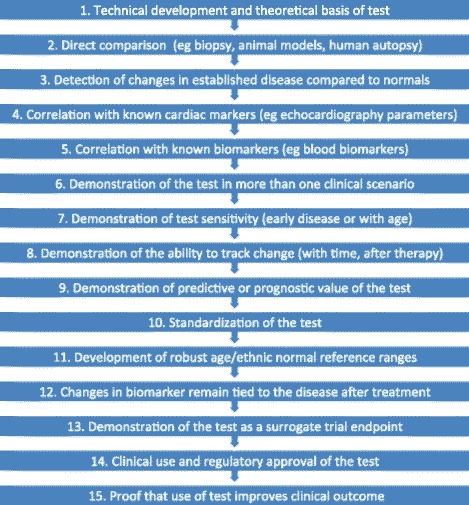
Roadmap for developing biomarkers derived from parametric mapping

Eventually, data on accuracy and prognosis will have to be followed by trials on the impact on patient outcomes. It is likely that relaxation times and ECV may achieve the status of biomarkers (Fig. 4), which allow for defining the current status of the myocardium [192].

## Conclusion

CMR mapping of T1, T2, T2* and ECV provides quantitative information on changes of magnetic tissue properties, which reflect alterations of myocardial tissue composition. CMR mapping methodology has left behind the early stages of principal implementation and validation, and robust techniques are available for commercial CMR systems. Iron overload, amyloidosis, Anderson-Fabry, and myocarditis are clinical scenarios where cardiac mapping provides unique information and should be applied to guide clinical care. Due to its additional diagnostic and prognostic value in the assessment of diffuse myocardial disease, parametric mapping should be considered in the diagnostic evaluation of all patients with heart failure.

This document provides recommendations related to both clinical indications and practical implementation, and summarizes the underlying rationale. Building on the 2013 Consensus statement on myocardial T1 mapping and extracellular volume quantification [2], which primarily provided guidance on technical aspects, the clinical focus of this document reflects the growing body of evidence regarding the clinical utility of this maturing field. Where they overlap, the recommendations of this document are in agreement with those of the previous consensus statement with the two exceptions that a waiting time of 10 min after contrast application for post-contrast T1 mapping is now regarded sufficient (formerly 15 min; see **T1 mapping/ ECV**) and that ECV should be given as a percentage (see **Terminology**).

CMR parametric mapping has seen many innovations over the last decade and continuous to attract interest from both basic and clinical researchers. Thus, any consensus document can only attempt to reflect the state of evidence at a single point in time, and will invariably start to be incomplete by the time of publication. Nevertheless, this document is meant to give guidance for CMR clinicians who would like to provide state-of-the-art tissue characterization for patients with myocardial disease.

## Abbreviations

3CH: 3-chamber view
4CH: 4-chamber view
AIR: Arrhythmia-insensitive-rapid
AL: Immunoglobulin light-chain derived amyloid
ANGIE: Accelerated and navigator-gated look-locker imaging for cardiac T1 estimation
ARVD: Arrhythmogenic right-ventricular dysplasia
ATTR: Transthyretin amyloid
B0: Constant magnetic field
B1: Radio frequency field
bSSFP: Balanced steady-state free precession
CMR: Cardiovascular magnetic resonance
ECG: Electrocardiogram
ECV: Extracellular volume fraction
Gd-DTPA: Gadolinium diethylenetriamine penta-acetic acid
GraSE: Gradient-spin-echo
GRE: Gradient echo
ICD: Implanted cardioverter defibrillator
LGE: Late gadolinium enhancement
LV: Left ventricle
LVEF: Left-ventricular ejection fraction
MOLLI: Modified Look-Locker inversion recovery
MT: Magnetization transfer
ROI: Region of interest
RV: Right ventricle
SAPPHIRE: Saturation pulse prepared heart-rate-independent inversion recovery
SAP-T1: Short acquisition period T1 mapping
SASHA: Saturation recovery single-shot acquisition
SAX: Short-axis orientation
ShMOLLI: Shortened modified Look-Locker inversion recovery
STIR: Short inversion time inversion recovery
STONE: Slice-interleaved T1 mapping
TSE: Turbo spin-echo
