# Correction to: Clinical recommendations for cardiovascular magnetic resonance mapping of T1, T2, T2* and extracellular volume: A consensus statement by the Society for Cardiovascular Magnetic Resonance (SCMR) endorsed by the European Association for Cardiovascular Imaging (EACVI)

**DOI:** 10.1186/s12968-017-0408-9

**Published:** 2018-02-07

**Authors:** Daniel R. Messroghli, James C. Moon, Vanessa M. Ferreira, Lars Grosse-Wortmann, Taigang He, Peter Kellman, Julia Mascherbauer, Reza Nezafat, Michael Salerno, Erik B. Schelbert, Andrew J. Taylor, Richard B. Thompson, Martin Ugander, Ruud B. van Heeswijk, Matthias G. Friedrich

**Affiliations:** 1 Department of Internal Medicine and Cardiology, Deutsches Herzzentrum Berlin, Berlin, Germany; 2 Department of Internal Medicine and Cardiology, Charité Universitätsmedizin Berlin, Augustenburger Platz 1, 13353 Berlin, Germany; 3 DZHK (German Centre for Cardiovascular Research), partner site Berlin, Augustenburger Platz 1, 13353 Berlin, Germany; 4 University College London and Barts Heart Centre, London, UK; 5 Oxford Centre for Clinical Magnetic Resonance Research, Division of Cardiovascular Medicine, Radcliffe Department of Medicine, University of Oxford, Oxford, UK; 6 Division of Cardiology in the Department of Pediatrics, The Hospital for Sick Children, University of Toronto, Toronto, ON Canada; 7 Cardiovascular Science Research Centre, St George’s, University of London, London, UK; 8 National Institutes of Health, Bethesda, MD USA; 9 Department of Internal Medicine II, Division of Cardiology, Vienna, Austria; 10 Department of Medicine (Cardiovascular Division), Beth Israel Deaconess Medical Center, Harvard Medical School, Boston, USA; 11 Departments of Medicine Cardiology Division, Radiology and Medical Imaging, and Biomedical Engineering, University of Virginia Health System, Charlottesville, VA USA; 12 Department of Medicine, University of Pittsburgh School of Medicine, Pittsburgh, PA USA; 13 UPMC Cardiovascular Magnetic Resonance Center, Heart and Vascular Institute, Pittsburgh, PA USA; 14 Clinical and Translational Science Institute, University of Pittsburgh, Pittsburgh, PA USA; 15 Department of Cardiovascular Medicine, Alfred Hospital and Baker Heart and Diabetes Institute, Melbourne, Australia; 16 Department of Biomedical Engineering, University of Alberta, Edmonton, Canada; 17 Department of Clinical Physiology, Karolinska Institutet, Karolinska University Hospital, Stockholm, Sweden; 18 Department of Radiology, Lausanne University Hospital (CHUV) and Lausanne University (UNIL), Lausanne, Switzerland; 19 Departments of Medicine and Diagnostic Radiology, McGill University, Montréal, Québec Canada; 20 Department of Medicine, Heidelberg University, Heidelberg, Germany; 21 Département de radiologie, Université de Montréal, Montréal, Québec Canada; 22 Departments of Cardiac Sciences and Radiology, University of Calgary, Calgary, Canada

## Correction to: J Cardiovasc Magn Reson (2017) 19: 75. DOI: 10.1186/s12968-017-0389-8

In the original publication this article was incorrectly published as a “Review article”, this has been corrected to a “Position paper”. Furthermore, in the original publication of this article [1] the “Competing interests” section was incorrect.

The original publication stated the following competing interests:

### Competing interests

The authors declare that they have no competing interests.

The corrected competing interest statement is as followed:

### Competing interests

Reza Nezafat has patents pending for methods for scar imaging in patients with arrhythmia, slice interleaved T1 and T2 mapping, and motion correction for parametric mapping, and receives patent royalties from Phillips Healthcare and Samsung Electronics.

Michael Salerno, Peter Kellman, Richard Thompson and Ruud B. van Heeswijk receive research support (source codes) from Siemens Healthineers.

Reza Nezafat receives research support (source codes) from Philips Healthcare.

Erik B. Schelbert has received MRI contrast material donated for research purposes from Bracco Diagnostics and has served on advisory boards for Bayer and Merck.

Martin Ugander states having Institutional research and development agreement regarding cardiovascular magnetic resonance with Siemens.

Matthias G. Friedrich is board member and consultant of Circle Cardiovascular Imaging Inc.

Vanessa M. Ferreira receives support by the National Institute for Health Research (NIHR) Oxford Biomedical Research Centre (BRC) and British Heart Foundation Centre of Research Excellence, Oxford.

Authors Daniel Messroghli, Lars Grosse-Wortmann, Taigang He, Julia Mascherbauer, James C. Moon and Andrew J. Taylor report no competing interests.
